# Visual memory and alcohol use in a middle-aged birth cohort

**DOI:** 10.1186/s12889-024-18153-1

**Published:** 2024-03-13

**Authors:** Atiqul Haq Mazumder, Jennifer H. Barnett, Anu-Helmi Halt, Marjo Taivalantti, Martta Kerkelä, Marjo-Riitta Järvelin, Juha Veijola

**Affiliations:** 1 Department of Psychiatry, Research Unit of Clinical Medicine, Faculty of Medicine, University of Oulu, Oulu, Finland; 2 Department of Psychiatry, Turku Psychosis and Substance Use (TuPSU), University of Turku, Turku, Finland; 3 Cambridge Cognition, University of Cambridge, Cambridge, UK; 4 Medical Research Centre Oulu, University Hospital of Oulu and University of Oulu, and Department of Psychiatry, University Hospital of Oulu, Oulu, Finland; 5 Center for Life Course Health Research, Faculty of Medicine, University of Oulu, Oulu, Finland; 6 Department of Epidemiology and Biostatistics, MRC Health Protection Agency (HPE), Centre for Environment and Health, School of Public Health, Imperial College London, London, UK; 7 Biocenter Oulu, University of Oulu, Oulu, Finland; 8 Unit of Primary Care, Oulu University Hospital, Oulu, Finland; 9 Department of Life Sciences, College of Health and Life Sciences, Brunel University London, Uxbridge, UK

**Keywords:** Alcohol use, Visual memory, Cognition, Middle age, Birth cohort

## Abstract

Light and moderate alcohol use has been reported to be associated with both impaired and enhanced cognition. The purpose of this study was to explore whether there was a linear relationship between visual memory and alcohol consumption in males and females in a large middle-aged birth cohort population in cross-sectional and longitudinal settings. Data were collected from 5585 participants completing 31-year (1997–1998) and 46-year (2012–2014) follow-ups including Paired Associate Learning (PAL) test at 46-years follow-up. The participants were originally from 12,231 study population of the Northern Finland Birth Cohort 1966 (NFBC1966). The PAL test was conducted to assess visual memory. Reported alcohol use was measured as total daily use of alcohol, beer, wine, and spirits converted into grams and as frequency and amount of use of beer, wine, and spirits. The total daily alcohol use was not associated with reduced visual memory. The frequency of use of beer and wine in males was associated with better visual memory in cross-sectional and longitudinal settings. Using six or more servings of spirits was associated with worse visual memory in males in cross-sectional and longitudinal settings. The study suggested a lack of a linear association between drinking and visual memory in the middle-aged population.

**Supplementary Information:**

The online version contains supplementary material available at 10.1186/s12889-024-18153-1.

## Introduction

In general population studies, both alcohol use disorder (AUD) and heavy drinking have been found to be associated with cognitive impairment, including visual memory deficits [1–5]. Alcohol use disorder (AUD) is characterized by clinically significant psychosocial and behavioural problems arising from repeated and continuous use of alcohol and episodic heavy drinking is referred to as having 4 or more drinks for females and 5 or more drinks for males in a single day in the past year [6, 7]. Visual memory is the capacity to remember what has previously been seen in the form of visual images [8]. Heavy episodic drinking has been found to be associated with impaired visual memory in both male and female adolescents [9]. Light and moderate drinking, on the other hand, have not been found to be associated with cognitive impairment. On the contrary, recent studies have found that light and moderate drinking are associated with cognitive enhancement [10–12]. Current light (mild) drinking is referred to as having at least 12 drinks in the past year but 3 drinks or fewer per week, on average over the past year. Current moderate drinking is referred to as having more than 3 drinks but no more than 7 drinks per week for women and more than 3 drinks but no more than 14 drinks per week for men, on average over the past year. Current heavier drinking is referred to as having more than 7 drinks per week for women: more than 14 drinks per week for men, on average over the past year. The weekly safe drinking limit is 14 units/ week for male and 7units/ week for female in the U.S.A. and 14 units/ week for both male and female in the U.K. [7, 13]. Daily ethanol consumption of ≥ 40 g for male or ≥ 20 g for females has been the modified criteria for hazardous (or risky) drinking [14]. Hazardous drinking is a pattern of alcohol consumption that increases the risk of harmful consequences for the user or other [15].

Cross-sectional studies mostly suggest that moderate to heavy drinking is associated with cognitive decline [16–19] and mild to moderate drinking is either associated with cognitive enhancement [19–23] or is not associated with cognitive performance [24, 25]. A dose-response positive association between amount of alcohol use and cognition has been found in females [26].

Longitudinal studies among the general population mostly suggest a positive correlation between light to moderate drinking and cognitive function [2, 27–30]. Some longitudinal studies, however, suggest no association, whether positive or negative [31–34]. Mild to moderate alcohol use, compared to former alcohol use and lifelong abstinence, has been found to be associated with better cognition [29].

There are not many longitudinal studies investigating the cognitive impact of alcohol use and any change in alcohol use in middle-aged people. So, it is interesting to know whether alcohol use is good or bad for cognition in middle-aged population both in cross-sectional and in longitudinal settings. From the clinician’s point of view, it is also important to know how different patterns and types of alcohol use affect cognition. In the current study, we focused on the potential linear relationship between visual memory and alcohol consumption (total daily use of alcohol converted into grams) in both cross-sectional and longitudinal settings in the Finnish middle-aged birth cohort population. The volume of alcohol can be converted into grams of ethanol using conversion calculator [14].

We also explored the association of different patterns (frequency and amount) and types of alcohol (beer, wine, spirits) with visual memory in the same population and in the same settings.

The main aim of this study was to explore the association of alcohol use with visual memory and new learning.

The specific research aims were to study the following:

1. The association of daily total alcohol use, converted into gram of ethanol, at 46 years of age with visual memory at 46 years of age (Cross-sectional study).
2. The association of grams per day alcohol use, converted into gram of ethanol, at 31 years with visual memory at 46 years of age (Longitudinal study 1).
3. The association of changes in daily total alcohol use, converted into gram of ethanol, from 31 to 46 years of age with visual memory at 46 years of age (Longitudinal study 2).
4. The association of the frequencies and amount of the use of beer, wine, and spirits with visual memory.

## Materials and methods

### Study population

**Fig. 1:**
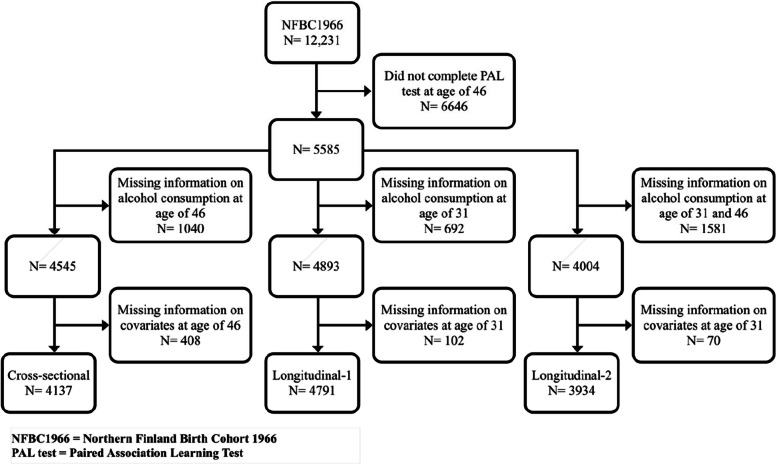
Flow chart showing selection of study population

The study population of the present study was selected from the participants of the Northern Finland Birth Cohort 1966 (NFBC1966) [35]. A total of 12,055 mothers with expected delivery dates between 1 January and 31 December 1966 from Oulu and Lapland (the two northernmost provinces of Finland) had been recruited for the study. Originally 12,231 males and females whose expected year of birth was 1966 were included in this study. Among them, 11,979 were born in 1966, 189 were born at the end of 1965 and 63 were born in early 1967. The follow-ups were conducted at 1, 14, 31, and 46 years of age [36]. Data from 5585 participants completing the 31-year (1997–1998) and 46-year (2012–2014) follow-ups and completing the Paired Associate Learning (PAL) test at 46-years follow-up were included in the present study (Fig. 1).

Information about alcohol consumption, background factors, and lifestyle were gathered using a postal questionnaire in 1997 when the participants were 31 years old. The survey was repeated, and the Paired Associative Learning (PAL) test was conducted, at follow-up in 2012–2014 when the participants were 46 years old.

From the original study population, 5,585 participants conducted the PAL test at the age of 46 years (Fig. 1).

The cross-sectional study exploring the association between alcohol use at the age of 46 years and cognition at the age of 46 years included 4,137 participants, after excluding those with missing information on alcohol consumption (*N* = 1,040) and covariates (*N* = 408) at the age of 46 years (Fig. 1).

Longitudinal study 1 exploring the association between alcohol use at the age of 31 years and cognition at the age of 46 years included 4,791 participants after excluding those with missing information on alcohol consumption (*N* = 692) and covariates (*N* = 102) at the age of 31 years (Fig. 1).

Longitudinal study 2 exploring the association between change in alcohol use between the ages of 31 years and 46 years and cognition at the age of 46 years included 3,934 participants after excluding those with missing information on alcohol consumption (*N* = 1,581) at the age 31 and 46, and covariates (*N* = 70) at the age of 31 years.

### Alcohol drinking measures

Alcohol use was measured by questionnaire at the ages of 31 years and 46 years. The items included questions on the frequency and amount of use of different types of alcohol (Beer and other light drinks, wine, and spirits).

One drink (alcohol dose) was also explained along with the questionnaire as follow:

- a bottle (33 cl) of medium beer or cider, or,
- a glass (12 cl) of mild wine or a small glass (8 cl) of fortified wine, or,
- a restaurant serving (4 cl) of spirits.

#### Calculation of gram per day alcohol use

Daily intake of total alcohol, as converted into grams of 100 % alcohol ethanol per day, daily intake of beer and other light drinks, in grams per day as converted into grams of 100 % ethanol, daily in-take of wine as converted into grams of 100 % ethanol, and intake of spirits, as converted into grams of 100 % ethanol in grams per day. We assumed one bottle of mild drink to contain one standard unit of alcohol (= 12 g of ethanol), wine to contain 13 % alcohol, and spirits to contain 38 % alcohol. From the questionnaire answers, daily consumption of ethanol was calculated by multiplying the typical frequency of use by quantity at both 31 years and 46 years.

#### Frequency of use of beer and other light drinks, wine, spirits

The frequency of alcohol use was measured on a 10-point scale (1 drinking never and 10 drinking daily). For the frequency of drinking light drinks, wine, or spirits, the cut-off was weekly drinking (drinking less frequently than once a week vs. drinking weekly or more frequently). Drinking less frequently than once a week covered drinking less than monthly and monthly. Drinking weekly or more frequently covered drinking weekly, daily or almost daily. We adopted this cut-off point from the frequency of binging question (Never, less than monthly, monthly, weekly, daily or almost daily) of the Alcohol Use Disorders Identification Test for Consumption (AUDIT-C) questionnaire. The AUDIT-C alcohol use questionnaire has been validated in Finland in previous studies [37, 38].

#### Amount of use of beer and other light drinks, wine, spirits

Quantities consumed on a typical occasion were asked about separately for all three beverage types: mild drinks in the number of 0.33 l bottles; wine in the number of glasses (= 12 cl) or 0.75 l bottles; and spirits in the number of 4 cl shots or 0.5 l bottles. The mild drink quantity was measured on a 9-point scale (1 drinking none and 9 drinking 15 bottles or more), and for wines and spirits, the typical quantity was measured similarly. The cut-off point for the amount was used when drinking for light drinks was 6 bottles (under 6 bottles vs. 6 bottles or more), and for wines, one bottle (under one bottle of wine vs. a bottle of wine or more), and for spirits six drinks (under six drinks vs. six drinks or more). We adopted this cut-off point from the binging question (How often do you have six or more drinks on one occasion) of the AUDIT-C questionnaire.

### Visual memory

The Paired Associative Learning (PAL) task from the Cambridge Neuropsychological Test Automated Battery (CANTAB) was used to assess visual memory. In the PAL test random patterns are shown in six boxes on the screen. The patterns are then displayed in the middle of the screen, one at a time, and the participant must select the box in which the pattern was originally located. PAL has been found to differentiate between Alzheimer’s disease and mild cognitive impairment [39]. PAL has been successfully used in different cultures and educations in both longitudinal and cross-sectional studies [40]. This study used the PAL test because it can reliably assess the type and degree of functional loss and the specificity of age-related cognitive decline [41].

In the PAL test, we assessed visual memory using the primary outcome variables, ‘total errors adjusted score’ and ‘first trial memory score’. Total Errors (Adjusted) (TEA) reflects how quickly the participant learns when they have multiple attempts at each problem, while First Trial Memory Score (FTMS) reflects how many patterns the participant correctly places on the first attempt at each problem.

Both PAL TEA and PAL FTMS were used as continuous variables.

### Covariates

For our cross-sectional analyses at the age of 46, we controlled for covariates those were collected at the same visit (at the age of 46), but for our longitudinal analyses, we controlled for baseline covariates collected at the earlier visit (at the age of 31) (Fig. 1). We used educational level, marital status, diet, physical activity, smoking and cardiometabolic diseases as covariates.

Educational level is associated with cognitive functioning [42]. The question addressing the education of the NFBC1966 participants was: ‘What is your basic education?’ (Less than 9 years of basic school, 9 years of basic school, matriculation examination). In the analysis, we combined ‘less than 9 years of basic school’ and ‘9 years of basic school’ as ‘No matriculation examination’.

The question addressing the marital status of the NFBC1966 participants was: ‘What is your relationship status?’ (Married since, cohabiting since, single, legal separation or divorced since, widowed since). In the analysis, we combined ‘married’ and ‘cohabiting’ as ‘In a relationship’, and ‘single’, ‘legal separation or divorced’ and ‘widowed’ as ‘Not in a relationship’.

Diet might be associated with cognitive functioning [43–45]. Diet was categorised as: ‘Healthy (Consuming vegetables, roots, and salad 3 times per week or more)’ and ‘Unhealthy’ (Consuming vegetables, roots, and salad 2 times per week or less)’.

Physical activity might [46–49] or might not [50, 51] be associated with cognition. Physical activity was categorised as: ‘Active (1 h or more brisk physical activity at a time causing at least some breathlessness and sweating)’ and ‘Inactive (Less than 1 h of brisk physical activity at a time causing at least some breathlessness and sweating)’.

Smoking has been found to be associated with reduced psychomotor speed in the middle age [52]. Heavy drinking with smoking has been associated with worse cognition than only heavy drinking [53, 54]. Heavy drinking with smoking has been associated with worse cognition than only heavy drinking [55]. Smoking has also been reported to exacerbate the cognitive decline associated with cardiometabolic multimorbidity [56]. Smoking was categorised as “Non-smoking” and “Smoking”.

Cardiometabolic diseases have long been recognised to be risk factors for cognitive decline and dementia [57–60]. Cognitive decline was reported to be accelerated in older adults with co-morbid cardiometabolic diseases like diabetes, hypertension, heart disease, and stroke [56]. Data on cardiometabolic diseases were collected from questionnaires used in 31-year and 46-year follow-ups. Cardiometabolic diseases were categorised as “Not having any cardiometabolic diseases” and “Having any cardiometabolic diseases”.

### Statistical analysis

Statistical analysis was performed using R version R 4.2.3. We evaluated the association between cognition and alcohol use using two different cognition variables: PAL FTMS and PAL TEA. The association between the PAL tests was analysed using linear regression, and a β with 95 % CI was reported. We considered < 0.01 *p*-value as statistically significant, as there were so many comparisons.

All continuous variables were normalised using z-scores. We assessed crude models and models adjusted with education, relationship status, diet, and physical activity. All analyses were conducted separately in males and females, as alcohol consumption differs between the two sexes [61] and performance in visual memory tests, including the PAL test, has also been reported to be different in males and females [62, 63]. Additionally, we performed multiple imputation by chained equations (MICE) on the drinking data, incorporating information on drinking habits and other covariates from both visits [64]. We also conducted sensitivity analyses using the imputed drinking and co-variate values.

### Attrition analysis

To evaluate the representativeness of our study population, an attrition analysis was performed while studying the associations with alcohol use in the longitudinal study 1 dataset.

## Results

### Background factors

About two-fifth of the participants in all three datasets were female. One-third of the males and more than half of the females had basic education above matriculation (12 years or more). About four-fifths of the participants were married or cohabitating. Half of the males and two-thirds of the females ate a healthy diet in both the cross-section at age 31 dataset and the change in alcohol use dataset. In the cross-section at age 46 dataset, three-fifths of males and four-fifths of females ate a healthy diet. One-third of males and one-fifths of females took active exercise in the age 31 and the change in alcohol use dataset. One-third of males and females took active exercise at the age of 46. Three-fourth of males and females were non-smokers in the age 31 and the change in alcohol use dataset. Three-fifth of males and females were non-smokers at the age of 46. Four-fifths of the participants did not have any cardiometabolic diseases (Table 1).

**Table 1:** Background factors for male and female participants in the cross-sectional, longitudinal 1, and longitudinal 2 datasets

Parameters	Cross-sectional		Longitudinal 1		Longitudinal 2	
	Male	Female	Male	Female	Male	Female
N	1877	2260	2053	2738	1747	2187
Education						
No matriculation (%)	1202 (64.0)	998 (44.2)	1339 (65.2)	1238 (45.2)	1120 (64.1)	978 (44.7)
Matriculation (%)	675 (36.0)	1262 (55.8)	714 (34.8)	1500 (54.8)	627 (35.9)	1209 (55.3)
Marital status						
Married / Cohabitating (%)	1530 (81.5)	1764 (78.1)	1481 (72.1)	2160 (78.9)	1268 (72.6)	1710 (78.2)
Single (%)	347 (18.5)	496 (21.9)	572 (27.9)	578 (21.1)	479 (27.4)	477 (21.8)
Diet						
Healthy (%)	1202 (64.0)	1813 (80.2)	1025 (49.9)	1860 (67.9)	899 (51.5)	1498 (68.5)
Unhealthy (%)	675 (36.0)	447 (19.8)	1028 (50.1)	878 (32.1)	848 (48.5)	689 (31.5)
Physical activity						
Active (%)	617 (32.9)	677 (30.0)	668 (32.5)	595 (21.7)	575 (32.9)	468 (21.4)
Inactive (%)	1260 (67.1)	1583 (70.0)	1385 (67.5)	2143 (78.3)	1172 (67.1)	1719 (78.6)
Smoking						
Non-smoker (%)	1299 (69.2)	1638 (72.5)	1578 (76.9)	1982 (72.4)	1334 (76.4)	1570 (71.8)
Smoker (%)	578 (30.8)	622 (27.5)	475 (23.1)	756 (27.6)	413 (23.6)	617 (28.2)
Cardiometabolic diseases						
No (%)	1389 (74.0)	1775 (78.5)	1754 (85.4)	2291 (83.7)	1494 (85.5)	1824 (83.4)
Yes (%)	488 (26.0)	485 (21.5)	299 (14.6)	447 (16.3)	253 (14.5)	363 (16.6)

Cross-sectional = Alcohol use data at the age of 46 and PAL test data at the age of 46

Longitudinal 1 = Alcohol use data at the age of 31 and PAL test data at the age of 46

Longitudinal 2 = Change in alcohol use data from the age of 31 to 46 and visual memory data at the age of 46

### Alcohol consumption and cognitive measures

In males, mean total alcohol consumption was 18 (SD 25) grams per day in the cross-sectional dataset and 13 (SD 18) in the longitudinal 1 dataset. In females, mean total alcohol consumption was 7 (SD 11) grams per day in the cross-sectional dataset and 5 (SD 10) in the longitudinal 1 dataset (Table 2).

**Table 2:** Alcohol consumption and visual memory for male and female participants in the cross-sectional, longitudinal 1, and longitudinal 2 datasets

Parameters	Cross-sectional		Longitudinal 1		Longitudinal 2	
	Male	Female	Male	Female	Male	Female
N	1877	2260	2053	2738	1747	2187
Total alcohol consumption (g/d) (mean (SD))	17.96 (24.97)	7.32 (11.75)	13.11 (17.77)	5.07 (9.17)	3.78 (21.16)	2.15 (11.31)
PAL TEA (mean (SD))	14.17 (14.01)	11.63 (9.97)	14.34 (14.29)	11.88 (10.67)	14.14 (14.09)	11.75 (10.28)
PAL FTMS (mean (SD))	19.09 (3.25)	19.56 (3.22)	19.05 (3.25)	19.53 (3.27)	19.10 (3.26)	19.54 (3.23)

Cross-sectional = Alcohol use data at the age of 46 and PAL test data at the age of 46

Longitudinal 1 = Alcohol use data at the age of 31 and PAL test data at the age of 46

Longitudinal 2 = Change in alcohol use data from the age of 31 to 46 and PAL test data at the age of 46

*PAL* Paired Association Learning, *TEA* Total Error Adjusted, *FTMS* First Trial Memory Score, *SD* Standard Deviation, *g/d* Grams per decilitre

The mean PAL TEA score was 14 in males and 12 in females in all datasets. The mean PAL FTMS score was 19 in males and 20 in females in all datasets (Table 2).

### Association of grams per day total alcohol use with visual memory

No statistically significant associations were found in males or females, between gram per day total alcohol use and visual memory in the cross-sectional, longitudinal 1 or longitudinal 2 settings (Table 3).

**Table 3:** Association of alcohol use in grams of alcohol per day with visual memory for male and female participants in the cross-sectional, longitudinal 1, and longitudinal 2 datasets

Parameters	PAL TEA				PAL FTMS			
	Crude		Adjusted		Crude		Adjusted	
	Beta (95% CI)	*p*	Beta (95% CI)	*p*	Beta (95% CI)	*p*	Beta (95% CI)	*p*
Males								
Cross-sectional	-0.008 (-0.052–0.037)	0.733	-0.016 (-0.06–0.028)	0.472	0.009 (-0.031–0.048)	0.663	0.016 (-0.023–0.055)	0.422
Longitudinal 1	-0.019 (0.055–0.025)	0.398	-0.033 (-0.077–0.011)	0.137	0.003 (-0.036–0.041)	0.898	0.018 (-0.021–0.056)	0.366
Longitudinal 2	-0.022 (-0.072–0.027)	0.373	-0.016 (-0.065–0.032)	0.51	0.032 (-0.011–0.076)	0.147	0.026 (-0.017–0.069)	0.238
Females								
Cross-sectional	-0.009 (-0.07–0.053)	0.776	-0.006 (-0.067–0.055)	0.847	0.043 (-0.033–0.119)	0.27	0.042 (-0.033–0.117)	0.271
Longitudinal 1	-0.033 (-0.088–0.022)	0.244	-0.018 (-0.073–0.037)	0.528	0.037 (-0.028–0.102)	0.26	0.024 (-0.041–0.089)	0.468
Longitudinal 2	0.036 (-0.024–0.096)	0.238	0.032 (-0.027–0.092)	0.29	0.001 (-0.071–0.073)	0.984	0.003 (-0.068–0.075)	0.924

Linear regression analysis. All continuous variables used in these models were normalised using z-scores. Adjusted models included education, marital status, diet, physical activity, smoking and cardiometabolic diseases

Cross-sectional = Alcohol use data at the age of 46 and PAL test data at the age of 46

Longitudinal 1 = Alcohol use data at the age of 31 and PAL test data at the age of 46

Longitudinal 2 = Change in alcohol use data from the age of 31 to 46 and PAL test data at the age of 46

*PAL* Paired Association Learning, *TEA* Total Error Adjusted, *FTMS* First Trial Memory Score, *CI* Confidence Interval

### Association of gram per day beer and other light drinks, wine, and spirits use with visual memory

No statistically significant associations were found in males or females, between gram per day beer and other light drinks, wine, and spirits use and visual memory in the cross-sectional or longitudinal 1 setting (Supplementary Tables 1–3).

### Association of frequency of beer and other light drinks, wine, and spirit use with visual memory in the cross-sectional and longitudinal 1 datasets

The association between frequency of use of beer and other light drinks, wine, spirits and visual memory in males and females in the cross-sectional and longitudinal 1 datasets are shown in Supplementary Tables 4–6.

Males using beer and other light drinks and other light drinks once a week or more at 46 years of age made less errors in visual memory task at the same age (cross-sectional) compared to males using beer and other light drinks less than once a week, both before and after adjustment. They also made higher scores in PAL test before adjustment. Males using beer and other light drinks once a week or more at 31 years of age also made less errors in visual memory task and higher scores in PAL test at the age of 46 years (longitudinal-1) compared to males using beer and other light drinks less than once a week, both before and after adjustment. However, none of these findings were statistically significant (Supplementary Table 4).

Males who used to drink wine once a week or more at the age of 46 years (cross-sectional) and those who used to drink wine once a week or more at the age of 31 years (longitudinal-1) both made less errors in visual memory task and higher scores in PAL test at the age of 46 years compared to their corresponding counterparts, before adjustment, with statistically significant difference in cross-sectional setting (Supplementary Table 5).

Females who used to drink spirits once a week or more at the age of 46 years made more errors and lower scores in PAL test at the same age (46 years, cross-sectional) compared to those who did not. But the differences were not statistically significant (Supplementary Table 6).

### Association of amount of beer and other light drinks, wine, and spirit use with visual memory in the cross-sectional and longitudinal 1 datasets

The association between amount of beer and other light drinks, wine, spirits and visual memory in males and females in the cross-sectional and longitudinal 1 datasets are shown in Supplementary Tables 7–9.

There appear to be no significant findings about the amount of use of beer and other light drinks and wine with visual memory (Supplementary Tables 7 and 8).

Males using six or more servings of spirits at 31 years made more errors before adjustment and lower scores before and after adjustment, in PAL test at 46 years (longitudinal 1) compared to males using fewer than six servings, and the differences were not statistically significant (Supplementary Table 9).

### Attrition analysis

For longitudinal study-1, alcohol use data at age 31 years were available for 2,744 male and 3,369 female participants. At the age of 46 years, 25 % of males and 18.7 % of females had dropped out. The dropped-out males used significantly more alcohol and beer and other light drinks than those who participated at the age of 46 years (Supplementary Table 10).

For cross-sectional study at the age of 45 years, alcohol use data were available for 2,747 male and 3,021 female participants. About 32 % of males and 25 % of females had dropped out. The dropped-out males used more alcohol and beer and other light drinks than those who didn’t (Supplementary Table 10).

### Sensitivity analyses in cross-sectional dataset

For our cross-sectional study, we have done sensitivity analyses in the cross-sectional dataset among male and female participants who were present at both visits (At the ages of 31 and 46 years). The results were like those of our original analyses (Supplementary Table 11).

### Sensitivity analyses using multiple imputation

We performed multiple imputation by chained equations (MICE) on the drinking data, incorporating information on different drinking patterns and covariates models included education, marital status, diet, physical activity, smoking and cardiometabolic diseases from both 31-year and 46-year follow-ups. Then we performed sensitivity analyses using the imputed drinking data and found no differences (Supplementary Table 12).

### Summary of results

In summary, we did not find any association of cognitive decline with daily use of total alcohol, beer and other light drinks, wine and spirits, converted into grams of ethanol, which was not as per our expectations. Females drinking spirits once a week or more cross-sectionally, performed significantly worse on PAL TEA and made less scores on PAL FTMS compared to those using less frequently. Males using six or more servings of spirits made less first trial memory scores longitudinally compared to those using less than six. Males using beer and other light drinks and wine once a week or more made less errors and higher scores in PAL test longitudinally compared to those using less frequently.

## Discussion

### Summary of main findings

Most participants reported light consumption of alcohol. Because there were not many differences, our study did not report many statistically significant findings. The main finding of our birth-cohort-based longitudinal population study revealed that in mid-life when controlled for education, marital status, diet, physical activity, smoking and cardiometabolic diseases, the daily use of total alcohol, beer and other light drinks, wine and spirits, converted into grams of ethanol, was not associated with decreased visual memory in both cross-sectional and longitudinal settings. Both wine and beer and other light drinks results for men are on same direction: Those who used to drink once a week or more at 31 years made less errors and higher scores in PAL test at 46 years (longitudinal 1) compared to the those who used to drink ‘Less than once a week’. Women using spirits once a week or more at the age 46 years made more errors and lower scores in PAL test at 46 years (cross-sectional) compared to those who did not. Men using six or more servings of spirits at 31 years made less first trial memory scores compared to those using less than six servings of spirits. Almost all these results were not statistically significant.

### Comparison with other studies

In general population studies, AUD has been found to be associated with visual memory deficits [1, 3]. Heavy drinking has also been found to be associated with impaired visual memory [2] and reaction time [4]. Heavy episodic drinking has also been found to be associated with poor visual memory in adolescents [9]. A positive association between moderate drinking and cognition has also been found [65]. In the present study, we found that use of six or more servings of spirits was associated with impaired visual memory in males, but that was the only finding that showed a negative association between drinking and cognition.

There are not many longitudinal studies investigating the cognitive impact of alcohol use and change in alcohol use in middle-aged people. In a study by Yan and colleagues no significant association of mild to moderate alcohol use with cognition was found in both the baseline and follow-up after 1 year [66]. A prospective cohort study of the middle-aged to elderly population with 9 years of mean follow-up revealed that mild to moderate alcohol consumption was associated with better cognitive functioning [67]. In another longitudinal study, an increase in alcohol use from the baseline to follow-up after 11 years was found to be associated with better cognition in females [30]. A fourth study by Arntzen et al. reported that light to moderate wine use at the baseline was associated with better cognition at follow-up after 7 years [68]. Our current study did not find any association between the baseline use of alcohol and cognition at the follow-up provided the fact that we did not measure cognition at all at the baseline.

As in our study, some other longitudinal studies, however, suggest no association, whether positive or negative [31–34]. One recent study also did not find any significant association of mild to moderate alcohol use with cognition compared to nonalcohol users both at the baseline and at follow-up after 1 year where age, education, smoking, tea drinking, hypertension, hyperlipidemia, diabetes and traumatic brain injury were used as covariates [66].

Earlier cross-sectional studies suggested that mild to moderate drinking was either associated with cognitive enhancement [19–23] or was not associated with cognition at all [24, 25]. The study findings in our cross-sectional dataset at age 46 were like those studies.

Hop-Derived Iso-α-Acid, a chemical component found in dark beer and other light drinks, has been associated with better visual memory in animal model studies [69]. Our finding on beer and other light drinks use did not comply with this finding, as we found a negative association between beer and other light drinks use frequency and visual memory. Resveratrol, a polyphenol compound found in red wine, has been associated with better cognitive performance in a double-blind, placebo-controlled, crossover study in humans [70]. This might explain our findings suggesting an association of frequency of wine consumption with better visual memory in males. However, white wine has little of this component, and there is no record of whether the wine consumed was, red, rose or white. The association between increased amount of spirits and impaired visual memory in males in our study can be explained by the activation of pro-inflammatory cytokines, free-radical damage, and thiamine deficiency caused by ethanol-induced neurotoxicity [71].

The number of drinks per week has been found to be associated with cognitive impairment [72]. However, one cross-sectional cohort study revealed that moderate, regular drinking was associated with better cognition than non-drinking and less-frequent drinking [20]. Another cohort study revealed that irregular drinking and abstinence were associated with cognitive decline compared to regular drinking [73]. In the cross-sectional part of our cohort study, more frequent use of wine was found to be associated with better cognition in males.

A dose-response positive association between alcohol use and cognition has been found among female current drinkers compared to former heavy drinkers and abstainers [26]. An increase in alcohol use from the baseline to follow-up after 11 years has been found to be associated with better cognition in females [30]. In our study, changes in the amount of alcohol use from baseline to follow up were not associated with cognitive function at follow up. We, however, did not measure cognition at the baseline. Light to moderate wine use at the baseline has been found to be associated with better cognition at follow-up after 7 years [68]. In our study, more frequent use of wine in males were associated with better cognition both cross-sectionally and longitudinally.

### Strengths

Different patterns of alcohol use in both cross-sectional and longitudinal settings of a large cohort population are the novel aspects of the current study. The age group of the participants was middle aged, which has not been studied thoroughly previously. We were able to use multiple covariates like education, marital status, diet, physical activity, smoking and cardiometabolic diseases in this study. Gram per day use of total alcohol, beers, wine, and spirits and frequencies and amounts of beer and other light drinks, wine and spirits were used. We were able to study the association between visual memory and reported alcohol use in both cross-sectional and longitudinal settings. The longitudinal setting included using both alcohol use at age 31 years and change in alcohol use between ages 31 and 46 years as exposures.

### Limitations

We did not categorise alcohol use into mild, moderate, and heavy; neither did we exclude abstinence. Most of the alcohol use measures were self-reported which might introduce biases. It is also important to note that our results did not represent cognition in general, but only one very limited aspect (visual memory) of this wide phenomenon. As only one cognitive measure was applied, we could not draw conclusions on general cognitive performance. No measure of cognition or visual memory was assessed at the baseline. Without such a baseline control, it is difficult to interpret the findings and the direction of the effect. Only educational attainment was used to assess socioeconomic status (SES), not household income although educational attainment largely correlates with earning [74, 75]. Higher SES participants are likely to have both better visual memory and better incomes to purchase wine. Education level was assessed only via a binary variable (no matriculation exam vs. matriculation exam). Depression is associated with impairment of memory [76] and executive functioning [77]. In the current study, however, we did not use depressive symptoms as a possible confounder because a recent study found no association between depressive symptoms and visual memory in the same population [78]. Possible changes in marital status, diet, and exercise over time might also make it difficult to predict the true effects of covariants in this longitudinal study. We did not present interaction analyses by sex to show whether any findings were indeed different for men and women, or whether the differences in the observed sex-specific associations between alcohol use and cognition stemmed from differences in statistical power. The high attrition level was another limitation of our study.

### Clinical and public health implications

It is important to study cognition and its relationship with alcohol, if alcohol use affects cognition and if cognition affects the use of alcohol. Our results suggest that light to moderate alcohol use might not be harmful for cognition in the general population. This might have an impact on public health, and the statutory bodies might need to reform their existing alcohol policies. However, the findings of this study should be interpreted cautiously, and no clinical conclusions can be drawn until the results of this study are replicated in other studies.

## Conclusions

It is important to study cognition and its relationship with alcohol, if alcohol use affects cognition and if cognition affects the use of alcohol. Our study suggests a lack of a linear association between reported alcohol use and visual memory. The findings of the current study concerning the association of reported alcohol use with cognitive enhancement in male middle-aged birth-cohort population both cross-sectionally and longitudinally are novel. Based on our study, recommendations on the safety limits of alcohol use in adults might need to be reconsidered. The observed gender-specific findings in our study are interesting and require further investigation. These might encourage future researchers to explore more in this field.

### Supplementary Information

**Supplementary Material 1.**

**Supplementary Material 2.**

## Data Availability

NFBC data is available from the University of Oulu, Infrastructure for Population Studies. Permission to use the data can be applied for research purposes via an electronic material request portal. In the use of data, we follow the EU general data protection regulation (679/2016) and Finnish Data Protection Act. The use of personal data is based on cohort participant’s written informed consent at his/her latest follow-up study, which may cause limitations to its use. Please, contact NFBC project center (NFBCprojectcenter@oulu.fi) and visit the cohort website for more information.
