# Supplementary material for: Visual memory and alcohol use in a middle-aged birth cohort
Source: BMC Public Health. 2024 Mar 13;24:788. doi: 10.1186/s12889-024-18153-1 (PMC10935933; doi:10.1186/s12889-024-18153-1)
Supplement: Supplementary file 1 — Supplementary Material 1. [file 12889_2024_18153_MOESM1_ESM.docx]

Association of daily beer and other light drinks use, converted into grams of ethanol with visual memory for male and female participants in the cross-sectional and longitudinal 1 datasets

| Parameters | PAL TEA | |  |  |  |  | PAL FTMS | |  |  |
| --- | --- | --- | --- | --- | --- | --- | --- | --- | --- | --- |
|  | Crude | | Adjusted | |  | Crude | | Adjusted | |  |
|  | Beta (95 % CI) | p | Beta (95 % CI) | p |  | Beta (95 % CI) | p | Beta (95 % CI) | p |  |
| Males |  |  |  |  |  |  |  |  |  |  |
| Cross-sectional | -0.011 (-0.055-0.032) | 0.618 | -0.024 (-0.067-0.019) | 0.271 |  | -0.008 (-0.047-0.031) | 0.685 | 0.004 (-0.035-0.042) | 0.855 |  |
| Longitudinal 1 | -0.023 (-0.065-0.02) | 0.291 | -0.036 (-0.078-0.006) | 0.092 |  | 0.001 (-0.036-0.038) | 0.94 | 0.014 (-0.023-0.05) | 0.461 |  |
| Females |  |  |  |  |  |  |  |  |  |  |
| Cross-sectional | 0.013 (-0.043-0.068) | 0.655 | -0.001 (-0.056-0.055) | 0.983 |  | 0.015 (-0.054-0.083) | 0.673 | 0.036 (-0.033-0.104) | 0.305 |  |
| Longitudinal 1 | -0.005 (-0.06-0.05) | 0.859 | -0.004 (-0.059-0.05) | 0.879 |  | 0.007 (-0.057-0.072) | 0.825 | 0.012 (-0.052-0.076) | 0.715 |  |
| Linear regression analysis. All continuous variables used in these models were normalised using z-scores. Adjusted models included education, marital status, diet, physical activity, smoking and cardiometabolic diseases.  Cross-sectional = Alcohol use data at the age of 46 and PAL test data at the age of 46.  Longitudinal 1 = Alcohol use data at the age of 31 and PAL test data at the age of 46.  PAL = Paired Association Learning. TEA = Total Error Adjusted. FTMS = First Trial Memory Score.  CI = Confidence Interval. | | | | | | | | | | |

Association of daily wine use, converted into grams of ethanol with visual memory for male and female participants in the cross-sectional and longitudinal 1 datasets

| Parameters | PAL TEA | |  |  |  |  | PAL FTMS | |  |  |
| --- | --- | --- | --- | --- | --- | --- | --- | --- | --- | --- |
|  | Crude | | Adjusted | |  | Crude | | Adjusted | |  |
|  | Beta (95 % CI) | p | Beta (95 % CI) | p |  | Beta (95 % CI) | p | Beta (95 % CI) | p |  |
| Males |  |  |  |  |  |  |  |  |  |  |
| Cross-sectional | -0.011 (-0.053-0.031) | 0.602 | 0.008 (-0.034-0.049) | 0.717 |  | 0.043 (0.006-0.081) | 0.023 | 0.026 (-0.011-0.063) | 0.174 |  |
| Longitudinal 1 | -0.049 (-0.098--0.001) | 0.046 | -0.035 (-0.083-0.013) | 0.152 |  | 0.064 (0.022-0.106) | 0.003 | 0.053 (0.012-0.095) | 0.012 |  |
| Females |  |  |  |  |  |  |  |  |  |  |
| Cross-sectional | -0.042 (-0.085-0.002) | 0.059 | -0.02 (-0.063-0.023) | 0.366 |  | 0.046 (-0.008-0.099) | 0.096 | 0.017 (-0.036-0.071) | 0.523 |  |
| Longitudinal 1 | -0.051 (-0.095--0.007) | 0.025 | -0.019 (-0.063-0.025) | 0.394 |  | 0.054 (0.002-0.106) | 0.042 | 0.017 (-0.035-0.068) | 0.532 |  |
| Linear regression analysis. All continuous variables used in these models were normalised using z-scores. Adjusted models included education, marital status, diet, physical activity, smoking and cardiometabolic diseases.  Cross-sectional = Alcohol use data at the age of 46 and PAL test data at the age of 46.  Longitudinal 1 = Alcohol use data at the age of 31 and PAL test data at the age of 46.  PAL = Paired Association Learning. TEA = Total Error Adjusted. FTMS = First Trial Memory Score.  CI = Confidence Interval. | | | | | | | | | | |

Association of daily spirits use, converted into grams of ethanol with visual memory for male and female participants in the cross-sectional and longitudinal 1 datasets

| Parameters | PAL TEA | |  |  |  |  | PAL FTMS | |  |  |
| --- | --- | --- | --- | --- | --- | --- | --- | --- | --- | --- |
|  | Crude | | Adjusted | |  | Crude | | Adjusted | |  |
|  | Beta (95 % CI) | p | Beta (95 % CI) | p |  | Beta (95 % CI) | p | Beta (95 % CI) | p |  |
| Males |  |  |  |  |  |  |  |  |  |  |
| Cross-sectional | 0.013 (-0.036-0.063) | 0.6 | -0.002 (-0.051-0.047) | 0.929 |  | 0 (-0.044-0.044 | 0.989 | 0.014 (-0.03-0.058) | 0.528 |  |
| Longitudinal 1 | 0.026 (-0.014-0.067) | 0.205 | 0.008 (-0.033-0.048) | 0.715 |  | -0.036 (-0.071-0) | 0.047 | -0.015 (-0.05-0.02) | 0.389 |  |
| Females |  |  |  |  |  |  |  |  |  |  |
| Cross-sectional | 0.065 (-0.069-0.199) | 0.339 | 0.077 (-0.055-0.209) | 0.253 |  | 0.023 (-0.143-0.189) | 0.784 | 0.009 (-0.154-0.172) | 0.912 |  |
| Longitudinal 1 | -0.032 (-0.101-0.036) | 0.354 | -0.034 (-0.102-0.033) | 0.32 |  | 0.045 (-0.036-0.126) | 0.274 | 0.049 (-0.031-0.128) | 0.227 |  |
| Linear regression analysis. All continuous variables used in these models were normalised using z-scores. Adjusted models included education, marital status, diet, physical activity, smoking and cardiometabolic diseases.  Cross-sectional = Alcohol use data at the age of 46 and PAL test data at the age of 46.  Longitudinal 1 = Alcohol use data at the age of 31 and PAL test data at the age of 46.  PAL = Paired Association Learning. TEA = Total Error Adjusted. FTMS = First Trial Memory Score.  CI = Confidence Interval. | | | | | | | | | | |

Association of frequency of beer and other light drinks use with visual memory for male and female participants in the cross-sectional and longitudinal 1 datasets

| Parameters | PAL TEA | |  |  |  |  | PAL FTMS | |  |  |
| --- | --- | --- | --- | --- | --- | --- | --- | --- | --- | --- |
|  | Crude | | Adjusted | |  | Crude | | Adjusted | |  |
| Frequency (Less than once a week as a reference) of beer and other light drinks use | Beta (95% CI) | p | Beta (95% CI) | p |  | Beta (95% CI) | p | Beta (95% CI) | p |  |
| Males |  |  |  |  |  |  |  |  |  |  |
| Cross-sectional | -0.166 (-0.268--0.063) | 0.003 | -0.13 (-0.232--0.029) | 0.012 |  | 0.101 (0.01-0.192) | 0.03 | 0.068 (-0.022-0.158) | 0.139 |  |
| Longitudinal 1 | -0.146 (-0.244--0.047) | 0.004 | -0.115 (-0.212--0.018) | 0.02 |  | 0.12 (0.034-0.205) | 0.006 | 0.091 (0.006-0.175) | 0.035 |  |
| Females |  |  |  |  |  |  |  |  |  |  |
| Cross-sectional | -0.057 (-0.125-0.01) | 0.095 | -0.046 (-0.113-0.021) | 0.175 |  | 0.088 (0.005-0.172) | 0.039 | 0.075 (-0.007-0.157) | 0.073 |  |
| Longitudinal 1 | -0.051 (-0.119-0.017) | 0.142 | -0.024 (-0.091-0.043) | 0.484 |  | 0.08 (0-0.161) | 0.05 | 0.048 (-0.031-0.127) | 0.225 |  |
| Linear regression analysis. All continuous variables used in these models were normalised using z-scores. Adjusted models included education, marital status, diet, physical activity, smoking and cardiometabolic diseases.  Cross-sectional = Alcohol use data at the age of 46 and PAL test data at the age of 46.  Longitudinal 1 = Alcohol use data at the age of 31 and PAL test data at the age of 46.  PAL = Paired Association Learning. TEA = Total Error Adjusted. FTMS = First Trial Memory Score.  CI = Confidence Interval. | | | | | | | | | | |

Association of frequency of wine use with visual memory for male and female participants in the cross-sectional and longitudinal 1 datasets

| Parameters | PAL TEA | |  |  |  |  | PAL FTMS | |  |  |
| --- | --- | --- | --- | --- | --- | --- | --- | --- | --- | --- |
|  | Crude | | Adjusted | |  | Crude | | Adjusted | |  |
| Frequency (Less than once a week as a reference) of wine use | Beta (95% CI) | p | Beta (95% CI) | p |  | Beta (95% CI) | p | Beta (95% CI) | p |  |
| Males |  |  |  |  |  |  |  |  |  |  |
| Cross-sectional | -0.169 (-0.295--0.043) | 0.009 | -0.041 (-0.171-0.088) | 0.534 |  | 0.261 (0.149-0.373) | <.001 | 0.144 (0.03-0.259) | 0.013 |  |
| Longitudinal 1 | -0.198 (-0.386--0.011) | 0.038 | -0.083 (-0.27-0.104) | 0.384 |  | 0.219 (0.056-0.382) | 0.009 | 0.113 (-0.049-0.276) | 0.172 |  |
| Females |  |  |  |  |  |  |  |  |  |  |
| Cross-sectional | -0.085 (-0.163--0.007) | 0.032 | -0.038 (-0.116-0.04) | 0.338 |  | 0.09 (-0.006-0.187) | 0.067 | 0.028 (-0.068-0.125) | 0.564 |  |
| Longitudinal 1 | -0.075 (-0.193-0.043) | 0.21 | 0.014 (-0.103-0.131) | 0.818 |  | 0.087 (-0.051-0.226) | 0.217 | -0.022 (-0.159-0.116) | 0.758 |  |
| Linear regression analysis. All continuous variables used in these models were normalised using z-scores. Adjusted models included education, marital status, diet, physical activity, smoking and cardiometabolic diseases.  Cross-sectional = Alcohol use data at the age of 46 and PAL test data at the age of 46.  Longitudinal 1 = Alcohol use data at the age of 31 and PAL test data at the age of 46.  PAL = Paired Association Learning. TEA = Total Error Adjusted. FTMS = First Trial Memory Score.  CI = Confidence Interval. | | | | | | | | | | |

Association of frequency of spirits use with visual memory for male and female participants in the cross-sectional and longitudinal 1 datasets

| Parameters | PAL TEA | |  |  |  |  | PAL FTMS | |  |  |
| --- | --- | --- | --- | --- | --- | --- | --- | --- | --- | --- |
|  | Crude | | Adjusted | |  | Crude | | Adjusted | |  |
| Frequency (Less than once a week as a reference) of spirits use | Beta (95% CI) | p | Beta (95% CI) | p |  | Beta (95% CI) | p | Beta (95% CI) | p |  |
| Males |  |  |  |  |  |  |  |  |  |  |
| Cross-sectional | 0.268 (-0.246-0.783) | 0.306 | 0.171 (-0.335-0.678) | 0.508 |  | -0.261 (-0.71-0.187) | 0.253 | -0.164 (-0.604-0.15) | 0.276 |  |
| Longitudinal 1 | -0.01 (-0.178-0.157) | 0.903 | 0.006 (-0.159-0.171) | 0.945 |  | -0.017 (-0.163-0.129) | 0.82 | -0.029 (-0.172-0.114) | 0.691 |  |
| Females |  |  |  |  |  |  |  |  |  |  |
| Cross-sectional | 0.84 (-0.145-1.534) | 0.018 | 0.792 (0.107-1.477) | 0.024 |  | -0.958 (-1.818--0.097) | 0.029 | -0.891 (-1.739--0.044) | 0.039 |  |
| Longitudinal 1 | -0.039 (-0.289-0.211) | 0.759 | -0.025 (-0.271-0.22) | 0.84 |  | 0.008 (-0.286-0.301) | 0.959 | -0.002 (-0.29-0.286) | 0.99 |  |
| Linear regression analysis. All continuous variables used in these models were normalised using z-scores. Adjusted models included education, marital status, diet, physical activity, smoking and cardiometabolic diseases.  Cross-sectional = Alcohol use data at the age of 46 and PAL test data at the age of 46.  Longitudinal 1 = Alcohol use data at the age of 31 and PAL test data at the age of 46.  CI = Confidence Interval. | | | | | | | | | | |

**Association of amount of beer and other light drinks use with visual memory for male and female participants in the cross-sectional and longitudinal 1 datasets**

| Parameters | PAL TEA | |  |  |  |  | PAL FTMS | |  |  |
| --- | --- | --- | --- | --- | --- | --- | --- | --- | --- | --- |
|  | Crude | | Adjusted | |  | Crude | | Adjusted | |  |
| Amount (less than 6 servings as a reference group) of beer and other light drinks use | Beta (95% CI) | p | Beta (95% CI) | p |  | Beta (95% CI) | p | Beta (95% CI) | p |  |
| Males |  |  |  |  |  |  |  |  |  |  |
| Cross-sectional | 0.099 (-0.033-0.23) | 0.14 | 0.014 (-0.118-0.147) | 0.832 |  | -0.057 (-0.174-0.06) | 0.34 | 0.022 (-0.095-0.14) | 0.713 |  |
| Longitudinal 1 | 0.076 (-0.048-0.201) | 0.229 | 0.017 (-0.108-0.142) | 0.791 |  | -0.104 (-0.213-0.004) | 0.059 | -0.045 (-0.154-0.064) | 0.416 |  |
| Females |  |  |  |  |  |  |  |  |  |  |
| Cross-sectional | 0.059 (-0.09-0.208) | 0.439 | -0.008 (-0.157-0.142) | 0.922 |  | -0.041 (-0.226-0.143) | 0.66 | 0.056 (-0.129-0.241) | 0.553 |  |
| Longitudinal 1 | 0.136 (-0.017-0.288) | 0.081 | 0.093 (-0.059-0.245) | 0.232 |  | -0.107 (-0.287-0.072) | 0.241 | -0.035 (-0.214-0.144) | 0.702 |  |
| Linear regression analysis. All continuous variables used in these models were normalised using z-scores. Adjusted models included education, marital status, diet, physical activity, smoking and cardiometabolic diseases.  Cross-sectional = Alcohol use data at the age of 46 and PAL test data at the age of 46.  Longitudinal 1 = Alcohol use data at the age of 31 and PAL test data at the age of 46.  PAL = Paired Association Learning. TEA = Total Error Adjusted. FTMS = First Trial Memory Score.  CI = Confidence Interval. | | | | | | | | | | |

**Association of amount of wine use with visual memory for male and female participants in the cross-sectional, and longitudinal 1 datasets**

| Parameters | PAL TEA | |  |  |  |  | PAL FTMS | |  |
| --- | --- | --- | --- | --- | --- | --- | --- | --- | --- |
|  | Crude | | Adjusted | |  | Crude | | Adjusted | |
| Amount (less than 6 servings as a reference group) of wine use | Beta (95% CI) | p | Beta (95% CI) | p |  | Beta (95% CI) | p | Beta (95% CI) | p |
| Males |  |  |  |  |  |  |  |  |  |
| Cross-sectional | 0.139 (-0.106-0.384) | 0.267 | -0.058 (-0.185-0.301) | 0.64 |  | -0.118 (-0.333-0.098) | 0.284 | -0.046 (-0.259-0.166) | 0.671 |
| Longitudinal 1 | -0.058 (-0.205-0.089) | 0.44 | -0.094 (-0.239-0.052) | 0.207 |  | 0.092 (-0.036-0.22) | 0.157 | 0.134 (0.008-0.261) | 0.037 |
| Females |  |  |  |  |  |  |  |  |  |
| Cross-sectional | -0.074 (-0.383-0.236) | 0.64 | -0.106 (-0.41-0.199) | 0.497 |  | -0.003 (-0.39-0.383) | 0.987 | 0.046 (-0.334-0.426) | 0.812 |
| Longitudinal 1 | 0.056 (-0.099-0.211) | 0.479 | 0.042 (-0.111-0.195) | 0.594 |  | -0.065 (-0.248-0.117) | 0.482 | -0.044 (-0.224-0.136) | 0.631 |
| Linear regression analysis. All continuous variables used in these models were normalised using z-scores. Adjusted models included education, marital status, diet, physical activity, smoking and cardiometabolic diseases.  Cross-sectional = Alcohol use data at the age of 46 and PAL test data at the age of 46.  Longitudinal 1 = Alcohol use data at the age of 31 and PAL test data at the age of 46.  PAL = Paired Association Learning. TEA = Total Error Adjusted. FTMS = First Trial Memory Score.  CI = Confidence Interval. | | | | | | | | | |

**Association of amount of spirits use with visual memory for male and female participants in the cross-sectional, and longitudinal 1 datasets**

| Parameters | PAL TEA | |  |  |  |  | PAL FTMS | |  |  |
| --- | --- | --- | --- | --- | --- | --- | --- | --- | --- | --- |
|  | Crude | | Adjusted | |  | Crude | | Adjusted | |  |
| Amount (less than 6 servings as a reference group) of spirits use | Beta (95% CI) | p | Beta (95% CI) | p |  | Beta (95% CI) | p | Beta (95% CI) | p |  |
| Males |  |  |  |  |  |  |  |  |  |  |
| Cross-sectional | 0.237 (-0.008-0.481) | 0.058 | 0.102 (-0.144-0.348) | 0.417 |  | -0.159 (-0.383-0.064) | 0.161 | -0.04 (-0.264-0.185) | 0.73 |  |
| Longitudinal 1 | 0.215 (0.052-0.378) | 0.01 | 0.118 (-0.047-0.282) | 0.161 |  | -0.241 (-0.379--0.103) | 0.001 | -0.142 (-0.281--0.004) | 0.044 |  |
| Females |  |  |  |  |  |  |  |  |  |  |
| Cross-sectional | -0.038 (-0.626-0.55) | 0.899 | -0.076 (-0.656-0.504) | 0.797 |  | 0.177 (-0.55-0.904) | 0.633 | 0.237 (-0.479-0.952) | 0.517 |  |
| Longitudinal 1 | -0.049 (-0.54-0.443) | 0.846 | -0.119 (-0.604-0.366) | 0.63 |  | -0.012 (-0.58-0.555) | 0.966 | 0.079 (-0.479-0.638) | 0.781 |  |
| Linear regression analysis. All continuous variables used in these models were normalised using z-scores. Adjusted models included education, marital status, diet, physical activity, smoking and cardiometabolic diseases.  Cross-sectional = Alcohol use data at the age of 46 and PAL test data at the age of 46.  Longitudinal 1 = Alcohol use data at the age of 31 and PAL test data at the age of 46.  PAL = Paired Association Learning. TEA = Total Error Adjusted. FTMS = First Trial Memory Score.  CI = Confidence Interval. | | | | | | | | | | |

Attrition analysis: Alcohol use at the age of 31 and 46 and visual memory at the age of 46.

| Participants | Study sample | Dropouts | P-value |
| --- | --- | --- | --- |
| Males | 2053 | 691 |  |
| Total alcohol g/d (mean SD) at 31 years | 13.11 (17.77) | 17.05 (26.53) | <0.001 |
| Beer g/d (mean SD) at 31 years | 8.36 (12.85) | 11.08 (17.24) | <0.001 |
| Wine g/d (mean SD) at 31 years | 1.76 (4.90) | 1.92 (7.84) | 0.539 |
| Light wine g/d (mean SD) at 31 years | 0.10 (0.33) | 0.22 (1.86) | 0.006 |
| Spirit g/d (mean SD) at 31 years | 2.89 (6.62) | 3.83 (9.56) | 0.004 |
| Females | 2738 | 631 |  |
| Total alcohol g/d (mean SD) at 31 years | 5.07 (9.17) | 5.47 (10.28) | 0.334 |
| Beer g/d (mean SD) at 31 years | 3.01 (6.43) | 3.05 (6.25) | 0.894 |
| Wine g/d (mean SD) at 31 years | 1.38 (3.46) | 1.68 (5.66) | 0.084 |
| Light wine g/d (mean SD) at 31 years | 0.15 (0.66) | 0.11 (0.38) | 0.135 |
| Spirit g/d (mean SD) at 31 years | 0.53 (2.53) | 0.63 (1.72) | 0.340 |
| Participants | Continue | Drop out | P-value |
| Males | 1877 | 870 |  |
| Total alcohol g/d (mean SD) at 46 years | 17.96 (24.97) | 21.02 (32.97) | 0.007 |
| Beer g/d (mean SD) at 46 years | 11.31 (18.24) | 13.27 (21.84) | 0.014 |
| Wine g/d (mean SD) at 46 years | 3.23 (8.08) | 3.00 (9.05) | 0.508 |
| Spirit g/d (mean SD) at 46 years | 3.41 (9.09) | 4.74 (16.71) | 0.007 |
| Females | 2260 | 761 |  |
| Total alcohol g/d (mean SD) at 46 years | 7.32 (11.75) | 7.64 (15.68) | 0.548 |
| Beer g/d (mean SD) at 46 years | 4.09 (9.28) | 4.01 (9.47) | 0.848 |
| Wine g/d (mean SD) at 46 years | 2.66 (5.08) | 2.61 (5.53) | 0.803 |
| Spirit g/d (mean SD) at 46 years | 0.57 (2.18) | 1.02 (6.33) | 0.003 |

SD= Standard Deviation, g/d= grams per decilitre.

Sensitivity analyses in the cross-sectional dataset among male and female participants who were present at both visits (At the ages of 31 and 46 years).

| Parameters | | | | Cross-sectional analysis with exclusion of the participants missing 31-year follow-up | | | | | | | |  | |  |  |  |
| --- | --- | --- | --- | --- | --- | --- | --- | --- | --- | --- | --- | --- | --- | --- | --- | --- |
|  |  |  |  | Male | | | | Female | | | p |  |  |  |  |  |
| N | | | | 1632 | | | | 2023 | | |  |  | |  |  |  |
| Education | | | |  | | | |  | | |  |  | |  |  |  |
| No matriculation (%) | | | | 1045 (64.0) | | | | 897 (44.3) | | | <0.001 |  | |  |  |  |
| Matriculation (%) | | | | 587 (36.0) | | | | 1126 (55.7) | | |  |  | |  |  |  |
| Marital status | | | |  | | | |  | | |  |  | |  |  |  |
| Married / Cohabitating (%) | | | | 1342 (82.2) | | | | 1582 (78.2) | | | 0.003 |  | |  |  |  |
| Single (%) | | | | 290 (17.8) | | | | 441 (21.8) | | |  |  | |  |  |  |
| Diet | | | |  | | | |  | | |  |  | |  |  |  |
| Healthy (%) | | | | 1063 (65.1) | | | | 1624 (80.3) | | | <0.001 |  | |  |  |  |
| Unhealthy (%) | | | | 569 (34.9) | | | | 399 (19.7) | | |  |  | |  |  |  |
| Physical activity | | | |  | | | |  | | |  |  | |  |  |  |
| Active (%) | | | | 541 (33.1) | | | | 619 (30.6) | | | 0.107 |  | |  |  |  |
| Inactive (%) | | | | 1091 (66.9) | | | | 1404 (69.4) | | |  |  | |  |  |  |
| Smoking | | | |  | | | |  | | |  |  | |  |  |  |
| Non-smoker (%) | | | | 1131 (69.3) | | | | 1474 (72.9) | | | 0.020 |  | |  |  |  |
| Smoker (%) | | | | 501 (30.7) | | | | 549 (27.1) | | |  |  | |  |  |  |
| Cardiometabolic diseases | | | |  | | | |  | | |  |  | |  |  |  |
| No (%) | | | | 1215 (74.4) | | | | 1590 (78.6) | | | 0.004 |  | |  |  |  |
| Yes (%) | | | | 417 (25.6) | | | | 433 (21.4) | | |  |  | |  |  |  |
| Total alcohol consumption (g/d) (mean (SD)) | | | | 17.35 (23.98) | | | | 7.35 (11.94) | | | <0.001 |  | |  |  |  |
| PAL TEA (mean (SD)) | | | | 14.17 (14.21) | | | | 11.71 (10.05) | | | <0.001 |  | |  |  |  |
| PAL FTMS (mean (SD)) | | | | 19.09 (3.24) | | | | 19.54 (3.22) | | | <0.001 |  | |  |  |  |
| Parameters | PAL TEA | |  | |  |  |  | | PAL FTMS | | | |  | |  |  |
|  | Crude | | Adjusted | | |  | Crude | | | Adjusted | | | | |  |  |
|  | Beta (95% CI) | p | Beta (95% CI) | | p |  | Beta (95% CI) | | p | Beta (95% CI) | | | p | |  |  |
| Males |  |  |  | |  |  |  | |  |  | | |  | |  |  |
| Total alcohol | | -0.035 (-0.085-0.016) | 0.176 | -0.041 (-0.091-0.009) | | 0.112 |  | 0.028 (-0.016-0.072) | | 0.216 | 0.033 (-0.011-0.076) | | | 0.143 | |  |
| Beer | | -0.025 (-0.074-0.024) | 0.315 | -0.037 (-0.085-0.011) | | 0.134 |  | 0.003 (-0.039-0.046) | | 0.88 | 0.014 (-0.028-0.056) | | | 0.522 | |  |
| Wine | -0.047 (-0.097-0.004) | 0.069 | -0.022 (-0.072-0.028) | | 0.388 |  | 0.067 (0.024-0.111) | | 0.003 | 0.044 (0.001-0.088) | | | 0.047 | |  |  |
| Spirits | -0.004 (-0.059-0.051) | 0.894 | -0.016 (-0.071-0.038) | | 0.558 |  | 0.013 (-0.035-0.061) | | 0.588 | 0.025 (-0.022-0.073) | | | 0.299 | |  |  |
| Females |  |  |  | |  |  |  | |  |  | | |  | |  |  |
| Total alcohol | | | 0.007 (-0.057-0.072) | 0.822 | 0.011 (-0.053-0.074) | | 0.743 |  | 0.01 (-0.069-0.089) | | 0.804 | 0.01 (-0.068-0.088) | | | 0.807 | |
| Beer | | | 0.028 (-0.03-0.086) | 0.351 | 0.015 (-0.043-0.073) | | 0.61 |  | -0.015 (-0.086-0.056) | | 0.676 | 0.005 (-0.066-0.076) | | | 0.889 | |
| Wine | -0.038 (-0.084-0.007) | 0.101 | -0.017 (-0.062-0.029) | | 0.471 |  | 0.034 (-0.021-0.09) | | 0.226 | 0.008 (-0.048-0.063) | | | 0.79 | |  |  |
| Spirits | 0.072 (-0.065-0.209) | 0.303 | 0.007 (-0.057-0.072) | | 0.212 |  | 0.011 (-0.053-0.074) | | 0.765 | 0.01 (-0.069-0.089) | | | 0.918 | |  |  |
| Linear regression analysis. All continuous variables used in these models were normalised using z-scores. Adjusted models included education, marital status, diet, physical activity, smoking and cardiometabolic diseases.  Cross-sectional = Alcohol use data at the age of 46 and PAL test data at the age of 46.  PAL = Paired Association Learning. TEA = Total Error Adjusted. FTMS = First Trial Memory Score.  SD= Standard Deviation, g/d= grams per decilitre. | | | | | | | | | | | | | | | |  |

Sensitivity analyses using multiple imputation of drinking based on the drinking information from other visits and other covariates.

| Parameters | PAL TEA | |  |  |  |  | PAL FTMS | |  |  |
| --- | --- | --- | --- | --- | --- | --- | --- | --- | --- | --- |
|  | Crude | | Adjusted | |  | Crude | | Adjusted | |  |
|  | Beta (95 % CI) | p | Beta (95 % CI) | p |  | Beta (95 % CI) | p | Beta (95 % CI) | p |  |
| Males |  |  |  |  |  |  |  |  |  |  |
| Cross-sectional | -0.005 (-0.048-0.039) | 0.837 | -0.012 (-0.056-0.031) | 0.557 |  | 0.01 (-0.028-0.048) | 0.615 | 0.015 (-0.022-0.053) | 0.425 |  |
| Longitudinal 1 | 0.00 (-0.041-0.041) | 0,995 | -0.015 (-0.056-0.026) | 0.465 |  | -0.012 (-0.047-0.024) | 0.520 | 0.004 (-0.031-0.039) | 0.829 |  |
| Longitudinal 2 | -0.006 (-0.055-0.044) | 0.818 | 0.00 (-0.049-0.049) | 0.997 |  | 0.024 (-0.018-0.065) | 0.261 | 0.019 (-0.022-0.059) | 0.360 |  |
| Females |  |  |  |  |  |  |  |  |  |  |
| Cross-sectional | -0.007 (-0.069-0.056) | 0.836 | -0.001 (-0.063-0.06) | 0.963 |  | 0.053 (-0.018-0.124) | 0.144 | .048 (-0.022-0.119) | 0.181 |  |
| Longitudinal 1 | -0.036 (-0.091-0.019) | 0.203 | -0.023 (-0.078-0.032) | 0.416 |  | 0.045 (-0.019-0.108) | 0.166 | 0.034 (-0.029-0.098) | 0.285 |  |
| Longitudinal 2 | 0.025 (-0.037-0.088) | 0.425 | 0.021 (-0.04-0.083) | 0.500 |  | 0.014 (-0.057-0.086) | 0.692 | 0.017 (-0.054-0.088) | 0.637 |  |
| Linear regression analysis. All continuous variables used in these models were normalised using z-scores. Adjusted models included education, marital status, diet, physical activity, smoking and cardiometabolic diseases.  Cross-sectional = Alcohol use data at the age of 46 and PAL test data at the age of 46.  Longitudinal 1 = Alcohol use data at the age of 31 and PAL test data at the age of 46.  Longitudinal 2 = Change in alcohol use data from the age of 31 to 46 and PAL test data at the age of 46. | | | | | | | | | | |
